# Minireview: Parabens Exposure and Breast Cancer

**DOI:** 10.3390/ijerph19031873

**Published:** 2022-02-08

**Authors:** Emily Hager, Jiangang Chen, Ling Zhao

**Affiliations:** 1 Department of Nutrition, University of Tennessee, Knoxville, TN 37996, USA; emily.nicole.hager@gmail.com; 2 Department of Public Health, University of Tennessee, Knoxville, TN 37996, USA

**Keywords:** paraben, breast cancer, endocrine-disruptive chemicals

## Abstract

There is increasing recognition that environmental exposure to chemicals, such as endocrine-disruptive chemicals (EDCs), contributes to the development of breast cancer. Parabens are a group of EDCs commonly found in personal care products, foods, and pharmaceuticals. Systemic exposure to parabens has been confirmed by the ubiquitous detection of parabens in human blood and urine samples. Although evidence from in vivo and epidemiological studies linking parabens exposure to breast cancer is limited, the current evidence suggests that parabens may negatively interfere with some endocrine and intracrine targets relevant to breast carcinogenesis. So far, most studies have focused on a single paraben’s effects and the direct modulating effects on estrogen receptors or the androgen receptor in vitro. Recent studies have revealed that parabens can modulate local estrogen-converting enzymes, 17β-hydroxysteroid dehydrogenase 1 and 2 and increase local estrogen levels. Also, parabens can crosstalk with the human epidermal growth factor receptor 2 (HER2) pathway and work with ER signaling to increase pro-oncogenic c-Myc expression in ER+/HER2+ breast cancer cells. Future studies investigating paraben mixtures and their crosstalk with other EDCs or signaling pathways both in vitro and in vivo in the context of breast cancer development are warranted.

## 1. Introduction

Breast cancer remains the second leading cause of cancer deaths (only second to lung cancer) in women in the US in 2021. It is estimated that more than 40,000 women will die from breast cancer in 2021 [1]. The average risk of a woman developing breast cancer at some time in her life is about 13% [1].

The risk factors for breast cancer have been identified and are grouped into non-modifiable (familial and hereditary) factors and modifiable (environmental) factors [2]. The familial risk factors include a family history of breast cancer, other breast diseases, or high breast density. Women with inherited mutations, specifically in *BRCA1* and *BRCA2,* may have a higher breast cancer risk. The genetic risks of breast cancer only account for approximately 5–10% of breast cancers, whereas 90% of breast cancer occurrence is thought to be environment-related [2]. The most commonly known modifiable risk factors for breast cancer are obesity, sedentary lifestyle, having a first child after 30, post-menopausal hormone and oral contraceptives use, and xenoestrogen exposure [3]. 

A growing body of evidence from animal and human studies links exposure to environmental chemicals with increased risks of developing breast cancer. Among them are endocrine-disrupting chemicals (EDCs) [4,5,6]. EDCs are exogenous chemicals that can interfere with endogenous hormone action with key characteristics, including activating or antagonizing hormone receptors or altering receptor expression and downstream signaling events in the sensitive cells [7]. The most common examples of EDCs are dioxins, polychlorinated biphenyls, bisphenol A, and parabens found in products such as plastics, detergents, foods, toys, cosmetics, or pharmaceuticals [8]. Parabens are 4-hydroxybenzoic acid alkyl esters. Emerging evidence has now revealed the endocrine-disrupting propensity of parabens based on their adverse impact on development and reproduction from both in vitro and in vivo studies [9,10]. In this review, we summarize the current advances in understanding paraben exposure in humans and the mechanisms by which parabens may increase breast cancer risks.

## 2. Human Parabens Exposure

Humans have continuous and repetitive exposure to parabens. Parabens, which act as preservatives to prevent the growth of microorganisms, are commonly absorbed or ingested daily from paraben-containing personal care products, such as lotions, deodorants, hair care products, shaving products, pharmaceuticals, textiles, clothes, and foods [11,12,13]. Parabens are distributed systemically once they enter the body through dermal or gastrointestinal absorption. Parabens have been detected in human tissues, blood, breast milk, placenta, and urine [14,15,16]. Among all the parabens that humans are exposed to, the four most common parabens detected in biological fluids are methylparaben (MP), ethylparaben (EP), propylparaben (PP), and butylparaben (BP) [11,15,17]. 

On the other hand, isobutyl (IsoBP), isopropyl (IsoPP), and benzyl (BzP) parabens are less commonly used in household products [18,19]. Parabens undergo hydrolysis by esterase to *p*-hydroxybenzoic acid in human skin, small intestine, and liver. The hydrolysis rate of esterase on parabens increases with the length of the paraben’s side chain; as a result, MP is the most abundant parabens found intact in biological fluids. It remains debatable whether the levels of esterase are sufficient to hydrolyze all dermally applied parabens and whether individual variations in esterase levels or exposure to esterase inhibitors could result in incomplete hydrolysis [20,21,22]. However, it is believed that systemic free paraben levels are largely from dermal exposure due to relatively less active esterases in the skin compared to those in the intestinal and liver. High concentrations of the free form of parabens have been detected within hours after whole-body application [15]. Intact parabens that survive first-pass metabolism can bind to human serum albumin, which protects parabens from hydrolysis, and therefore facilitates greater accessibility of the free form of parabens to other tissues and the potential for bioaccumulation [14,23]. 

The major exposure of parabens in humans is believed to be through personal care products. MP, PP, EP, and BP (free and conjugated) have been detected in urine in 99.1%, 92.7%, 42.4%, and 47% of young children in the US, respectively, while comparable frequencies of each paraben were detected in the adult population [24,25]. The estimated total intake of parabens via personal care product usage ranges from 31–766 µg/kg body weight, with infants being at the upper end [11]. Humans can also be exposed to parabens through foods. The estimated total intake of parabens from foods is between 307–940 ng/kg body weight. The daily intake varies depending on the life stage, with infants having the highest total intake [11]. In addition, urine concentrations of parabens is considerably higher in women (17–237 ng/mL) compared to men (0.5–79.1 ng/mL) [17]. The levels of parabens in the human body seem to correlate with the amount and the frequency of exposure [26,27,28]. 

## 3. Parabens Exposure and Breast Cancer

Like many other EDCs, paraben exposure has been associated with poor reproductive health in women [29]. In a recent study, urinary parabens concentrations were associated with earlier breast development, pubic hair development, earlier menarche in girls, and earlier genital development in boys [30]. 

It has been postulated that exposure to some EDCs leads to exacerbated cell proliferation and tumor growth in the breasts [2,4,31]. To establish links between parabens and breast cancer, it is imperative to demonstrate the presence and localization of parabens within the breast tissue. Paraben levels in breast tissue extracted from axilla to sternum have been investigated [11,32]. MP was detected at approximately 16.6 ng per gram of the breast tissue, higher than previously documented [19]. The tissue level of PP was 16.8 ng/g, higher than MP. Other parabens, such as BP, EP, and IsoBP, were also detected, although at relatively lower levels [11]. However, no significant correlation was identified between the concentration of parabens and the location of breast tumors or the estrogen receptor status in the breast tumors. It has been shown that parabens have greater bioaccumulation within the outer areas (axilla and lateral) of the breast than the inner areas (mid and medial) [32]. The outer areas of the breast consist of more adipose tissue where the mammary glands are centrally located. Moreover, the detection and accumulation of parabens in the adipose tissue of the breast have been reported, possibly due to their moderate hydrophobicity [23]. Higher levels of parabens detected in the outer regions of the breasts might be associated with how deodorants are applied [32]. 

Perhaps due to the vast amount of exposure variability and weak estrogenicity of parabens (reviewed in more details below) compared to 17-β Estradiol, epidemiological evidence directly linking parabens exposure with breast cancer is still limited. In one study, the association between the circulating level of xenoestrogens and mammographic breast density, a powerful risk factor for breast cancer, was investigated in post-menopausal women. No association was found between serum levels of BP or PP and the density of the breasts [33,34]. In a recent case-control study, paraben levels in spot urine samples from 711 women with breast cancer were analyzed and compared with the 598 women without breast cancer [35]. It was found that the highest quintiles of urinary MP (414–3174 μg/g creatinine), PP (28–3116 μg/g creatinine), and total parabens (551–3766 μg/g creatinine) were associated with increased risks of breast cancer and all-cause mortality [35]. In addition, the associations were affected by body mass index (BMI). Associations for breast cancer incidence were more pronounced among women with lower BMI (BMI < 25.0 kg/m^2)^ than among women with higher BMI (BMI ≥ 25.0 kg/m^2^). In contrast, associations for mortality were shown to have an opposite trend with BMI [35]. In another case-control study with a multiethnic cohort, urinary parabens and other EDCs in 1032 post-menopausal women with breast cancer with different ethnic backgrounds and 1030 individually matched controls were analyzed [36]. Breast cancer risks in this multiethnic population were weakly inversely associated with paraben exposure [36]. It should be noted that prolonged daily exposure to a paraben mixture (i.e., more than one type of parabens), bioaccumulation, and interactions with other xenoestrogens can invoke local intracrine, paracrine, or endocrine changes, contributing to breast carcinogenesis [37]. Therefore, future investigations on the association of parabens with breast cancer are needed. Table 1 summarizes the strengths and limitations of human studies on parabens exposure and breast cancer.

## 4. Estrogenic Effects of Parabens

Despite the limited epidemiological evidence linking paraben exposure with breast cancer, recent in vitro and animal studies have shed light on the endocrine-modulating effects of parabens, suggesting that parabens may be implicated in breast carcinogenesis.

Estrogen is the primary sex hormone responsible for mammary gland development during critical life stages [38,39]. The pubertal surge of secretion of estrogen and progesterone from the ovaries stimulates proliferation of the myoepithelial cells within ducts [40,41]. In addition, estrogen induces growth and branching of mammary ducts in cooperation with other growth factors by activating the estrogen receptors (ER) located in the epithelium and stromal cells [42,43,44]. 

The estrogenic activity of parabens has been well documented and characterized [2,10,45,46,47,48,49,50]. Parabens have been shown to regulate ER-mediated gene expression in vitro [2,45,46,47,48]. MP, EP, PP, BP, and BzP have an estradiol equivalence factor (EEF) of 1.25 × 10^−7^, 6.48 × 10^−7^, 2.39 × 10^−6^, 8.09 × 10^−6^, and 1.35 × 10^−5^ respectively [13]. In silico simulation has shown that MP and EP interact with the agonist-binding pocket of human ERα. MP and EP bound to Glu353 and Arg394 of human ER with binding energies of −49.35 and −53.38 kcal/mol, respectively. Both Glu353 and Arg394 are located at the ligand-binding pocket of the ER known to play a critical role in human ERα–17β-estradiol (E2) interaction [49]. The values of the negative binding energy indicate that the binding of parabens to ERα is likely to be a spontaneous process. The stimulatory effects on the proliferation of breast cancerous MCF-7 and noncancerous MCF-10A cells in response to either a single or repetitive exposure to MP, PP, or BP were studied [51]. While all tested parabens increased cell proliferation of MCF-7 cells, only MP and BP at low doses increased MCF-10A cells proliferation after a single exposure, but not repeated exposure. Moreover, MP, PP, and BP exposure at low doses significantly increased E2 secretion in MCF-7 cells, but they all had the opposite effect on MCF-10A cells [51]. Table 2 summarizes the differential effects of parabens on the gene expression of ER and progesterone receptor (PR) in MCF 7 vs. MCF-10 A cells [52]. Interesting, the effect of parabens on ERα and ERβ can be effectively blocked by ICI 182,780, the ER antagonist, in the MCF-7 cells [52]. In contrast, co-treatment with ICI 182,780 did not attenuate the stimulatory effect of parabens on either ERα or ERβ in the MCF-10A cells [52]. ERα and ERβ seem to play opposing roles in regulating growth and differentiation in response to estrogens in the breasts [53,54]. PR, an estrogen-regulated protein, is among the most important prognostic and predictive markers of response to endocrine therapies in breast cancer patients [55]. PR acts as a critical factor in the induction, progression, and maintenance of the neoplastic phenotype of ER-positive breast cancer [56]. Future studies are warranted to understand the biological implications of differential actions of parabens on ERs and PR in cancerous vs. noncancerous breast epithelial cells.

The differential effect of parabens on the expression of genes related to cell cycle control and apoptosis has been investigated in MCF-7 and MCF-10A cells [57]. As shown in Table 3, these results further demonstrated variabilities in gene expression between cancerous and noncancerous breast epithelial cell lines in response to parabens. The discrepancy in cellular behavior between cancerous and noncancerous breast epithelia in response to parabens may suggest that parabens’ effects could be cell type-specific.

Global changes in gene expression profiles in response to parabens or E2 treatment have been studied in MCF-7 cells [58]. MCF-7 cells treated for 3 days with EP, PP, and BP, but not MP, showed gene expression profiles that correlated well with those genes in response to E2 treatment and the length of the alkyl chain of parabens was crucial for the estrogen-like profiles [58]. MCF-7 cells were also treated with 5 × 10^−4^ M MP, 10^−5^ M BP, or 10^−8^ M E2 for 7 days and the global gene expression profiles in response to the treatments were compared [59]. Of the 19,881 human genes investigated, 1972 genes were altered by ≥2-fold by MP, 1,292 genes were altered by ≥2-fold by BP, and 857 genes were altered ≥2-fold by E2. Only 61 genes were upregulated ≥2-fold by MP, BP and E2, while 198 genes were downregulated by ≥2-fold by all three test compounds [59]. Therefore, the majority of genes did not follow the same pattern of regulation by all three treatments in MCF-7 cells [59]. These results demonstrated that although parabens are estrogenic, their mimicry of E2 in global gene expression patterns is not perfect. Differences in gene expression profiles in cells in response to parabens could lead to different biological and developmental trajectories compared to the cells exposed to E2 [59]. 

In vivo studies of the effects of parabens on dynamic histological changes in mammary gland development have been studied in Sprague-Dawley rats [60]. Compared to the rats treated with the vehicle control, MP at doses that mimic human exposure levels during perinatal, prepuberty, and puberty significantly decreased amount of adipose tissue while increased expansion of the ductal tree and/or elevated amount of glandular tissue with a higher degree of branching. The authors identified puberty as the window of heightened sensitivity to MP, with increased glandular tissue and differential expression of 295 genes with significant enrichment of genes involved in DNA replication and cell cycle regulation [60]. It is worth noting that the genes modified by MP are also well represented in the gene signature profiles of human breast cancer, suggesting a possible link between MP exposure during the critical window of breast development and a higher risk of breast cancer [60]. 

In vivo studies of the effects of parabens on breast cancer initiation and progression are still scarce. In one study, MP at the levels that are commonly used in personal care products promoted the development of larger tumors than the placebo treatment in the nude mice xenografted with ER+ MCF-7 cells. Similarly, MP treatment significantly increased the volume of human patient-derived xenograft breast tumors compared to the placebo [61]. Additional in vivo studies using different breast cancer animal models are necessary to understand the full extent of the effects of parabens on breast cancer development.

## 5. The Nongenomic Activity of Parabens

In addition to nuclear receptors ERα and ERβ, membrane-bound G-protein-coupled estrogen receptor (GPER, GPR30 or GPER1) also binds to estrogens and activates downstream signaling events [62,63]. GPER acts through protein-kinase cascade, such as ERK1/2, and is involved in various physiological and pathological processes, including cancer progression [64,65]. GPER signaling regulates matrix metalloproteinases (MMPs), which are a group of enzymes that helps rearrange the tissue microenvironment and enable cancer metastasis [66,67,68,69]. GPER is highly expressed in cancerous breast epithelial cells and tumors, especially in those that are less responsive to ERα inhibitors treatment [63]. A three-dimensional (3D) culture of immortalized MCF-12A (ERα+, ERβ+, and GPER+) was used to model the features and behavior of the breast epithelium in vivo [70]. Treatment with PP at 10 µM for 16 days resulted in deformed acini and filling of the acinar lumen of the mammospheres, which could be reversed by both ER and GPER inhibitors [70]. In contrast, another study examined the elongation of MRC5 cells, an immortalized human lung fibroblast cell line expressing GPER, but not ERα, in response to parabens [71]. GPER agonists induce MRC5 cell elongation. However, neither MP (0.1 µM) nor EP (1.0 µM) showed GPER agonist activities in these cells [71]. Taken together, these results suggest that classical nuclear estrogen receptors might be required for parabens to act through GPER [71]. 

## 6. Intracrinology: Aromatase, 17 β-Hydroxysteroid Dehydrogenases, and Parabens

It is recognized that it is not just circulating levels of estrogens that matter. The local microenvironment affected by estrogens produced in situ through the intracrine mechanism is also critical [72,73]. Aromatase is involved in converting androgen to estrogen and contributes to the intracrine pathway that stimulates the growth of normal breast epithelial cells and cancer cells. Treatment of MP or PP induced aromatase (CYP19A1) mRNA and protein expression in MCF-7 cells [51]. Consistently, parabens increased estradiol production in MCF-7 cells [51]. In contrast, MP, PP, and BP decreased CYP19A1 mRNA and aromatase protein expression in noncancerous MCF-10A cells, resulting in decreased estradiol production in MCF-10A cells. Moreover, it was reported that MP, EP, PP, BP, isoPP, and BzP dose-dependently inhibited aromatase activity in human placenta-derived microsomes [74]. Aromatase expression is tissue-specific and tightly controlled by local signals activating alternative promoters in a timely and cell-specific manner [75,76]. Therefore, the discrepancy of the findings between the two studies [51,74]. suggests that parabens may modulate aromatase expression in a cell- and tissue-specific manner.

In post-menopausal women, the ovarian source of estrogens is greatly diminished. Consequently, the primary source of active estrogen in breast tissue is local steroid conversion. In breast tissue, 17β-hydroxysteroid dehydrogenases (17β-HSD1 and 17β-HSD2) regulate the local balance between potent and weak estrogens. 17β-HSD1 catalyzes the reduction of 17-ketosteroids and is responsible for converting the weak estrogenic estrone (E1) into E2; 17β-HSD2, on the other hand, oxidizes 17β-hydroxysteroids and decreases the local concentrations of strong estrogen E2 [77]. A high 17β-HSD2 to 17β-HSD1 ratio positively correlates with the survival of ERα-positive breast cancer patients [77,78,79]. The relative expression of the 17β-HSDs, combined with the availability of substrates, was shown to determine the final biological impact of the sex steroids on the local tissue [80]. It has been reported that MP, EP, PP, IsoPP, BP, IsoBP, phenylparaben (PhP), hexylparaben (HeP), BzP, and heptylparaben (HepP) but not the common metabolite *p*-hydroxybenzoic acid, inhibited 17β-HSD2 expression at 20 µM [81]. Parabens also size-dependently inhibited 17β-HSD1 with HexP and HepP having the most potent inhibition, whereas MP and EP had no effects on 17β-HSD1 [81]. MP, EP, and PP are smaller parabens and are among the most commonly used parabens compared to their larger counterparts; therefore, 17β-hsd2 inhibition is more likely to occur and is physiologically more relevant than 17β-HSD1 inhibition by parabens [81]. Moreover, smaller parabens, such as EP, have a more efficient inhibition of 17β-HSD2 with an IC_50_ in the low micromolar range [81], than their binding to ER with an EC_50_ in the millimolar range [82]. Therefore, the inhibition of 17β-HSD2 and the subsequently elevated local estradiol concentrations by smaller parabens are more likely to disturb the regulation of estrogenic target genes than their direct action through binding to ERs [81]. These results suggest that the estrogenic action of parabens should not be simply evaluated based on their ER binding capabilities. The modulations of key intracrine enzymes and the synergistic or additive inhibitory effects of paraben mixtures on 17β-HSDs should be investigated to provide a complete picture of parabens’ effects on the local tissue environment.

## 7. Antiandrogenic Effects of Parabens

The alternation of endogenous androgen levels also plays an important role in breast cancer development. A high level of circulating testosterone increases the breast cancer risk in pre-menopausal women. Also, androgen level is a strong prognostic factor for local relapse, contralateral breast cancer, and distant metastases in post-menopausal women [83,84,85,86]. Androgen treatment inhibited cell proliferation and increased apoptosis of some breast cancer cell lines but stimulated proliferation of other breast cancer cells [87,88,89]. In HEK293 cells transfected with androgen receptor (AR), parabens (MP, BP, and PP) at 10 μM inhibited the transcriptional activity of testosterone by 40%, 33%, and 19%, respectively [20]. Similarly, the antiandrogen activities of IsoBP, BP, isoPP, and PP were reported using CHO-K1 cells stably transfected with AR [90]. In this study, no antiandrogenic activities were detected for EP and MP [90]. In contrast, in MDA-kb2 cells transfected with MMTV-driven luciferase, moderate antagonizing effects of MP and EP against dihydrotestosterone (DHT) were detected while PP, BP, [91]. or BzP showed no antiandrogenic activity [92]. These results may reflect different cell models used in the studies. Naïve HEK-293 and CHO-K1 lack endogenous ARs, while MDA-kb2 cells express both endogenous AR and glucocorticoid receptors (GRs). Compounds that interfere with either receptor are expected to affect the MMTV-driven luciferase activities in the MDA-kb2 cells transfected with either reporter. Parabens, BP and BzP in particular, are known to have GR-agonist activities [93]. Therefore, the GR-mediated luciferase activity induced by BP and BzP could have obscured their inhibition of DHT-mediated luciferase activity.

## 8. Crosstalk with Human Epidermal Growth Factor Receptor 2 Pathway by Parabens

Parabens have been shown to crosstalk with other cell signaling pathways in breast cancer development. Human epidermal growth factor receptor 2 (HER2) is a transmembrane protein of the epidermal growth factor receptor family. Autophosphorylation of tyrosine residues upon receptor dimerization activates and initiates a cascade of cell signaling events, including PI3K/Akt/mTORC2, which regulates cell proliferation, malignant transformation, and tumorigenesis [94,95,96]. Approximately one-fourth of breast cancers overexpress HER2 [97], which is a negative prognostic indicator. The interplay between HER2 and ER signaling pathways in breast cancer is considered multidirectional and complex [98,99]. 

Pan and colleagues have investigated the interaction of parabens with breast cancer-related signaling pathways [99]. Both PP and BP effectively increased pro-oncogenic *c-Myc* mRNA expression in BT-474 cells (ER+/HER2+). The increase in *c-Myc* mRNA by PP and BP was further enhanced by heregulin (HRG), a HER2 ligand [99]. Co-treatment with HRG and parabens significantly increased c-Myc protein expression in BT-474 cells. The combination of BP and HRG synergistically stimulated the proliferation of BT-474 cells compared with the effects of BP alone, and the synergy was abolished by classic ER antagonists [99]. 

In contrast, there was no synergy on c-Myc protein expression in ERα negative SKBR3 cell line (ERα-/HER2+). These results indicate that the synergy between HRG and BP is ERα dependent [99]. Intriguingly, in MCF-7 cells, which are ERα positive but HER2 negative, HRG and BP co-treatment also significantly enhanced the induction of c-Myc protein [99]. These findings suggest that other signaling pathways from the HER family may also involve in the effects of HRG on the estrogenic effect of parabens in breast cancer cells. Moreover, these findings underscore the urgent need to re-assess the “weak” estrogenic effects of parabens, particularly at environmentally achievable doses, in the presence of other factors that crosstalk and act in concert with ER signaling pathways to modulate breast cancer cell growth and development.

## 9. Anchorage Independence, Migration, and Parabens

Increased cell proliferation is not the only hallmark of cancerous cells. The ability of cells to grow from anchorage-dependent to anchorage-independent predicts the tendency to form tumors in vivo [100]. It was reported that MP, BP, and PP induced anchorage-independent growth of non-transformed breast epithelial MCF-10A cells in semi-solid suspension culture, which has provided additional evidence to link parabens in breast carcinogenesis [100]. Over time, the cancerous epithelial cells can acquire the attributes of mesenchymal cells through a dedifferentiation process, namely, epithelial-to-mesenchymal transitions (EMT) [101,102,103]. Once breast cancer cells have undergone EMT, they can function as mesenchymal cells to migrate through the basement membrane and into circulation, leading to metastasis. A study has indicated that long-term exposure (20 ± 2 weeks) of MCF-7 cells to MP, PP, and BP increased migration as measured by scratch assays and with time-lapse microscopy [104]. Long-term MP, PP, and BP treatment also increased the invasive properties of MCF-7 cells as measured by matrix degradation assays and migration through Matrigel [104]. Further analysis indicated that parabens downregulated cell adhesion molecule E-cadherin and β-catenin in the MCF-7 cells. Similar results were obtained from another two other estrogen responsive cell lines T-47-D and ZR-75-1 [104]. These results suggest that parabens may promote EMT in the cancerous breast epithelial cells.

## 10. Conclusions and Future Perspectives

**Figure 1:**
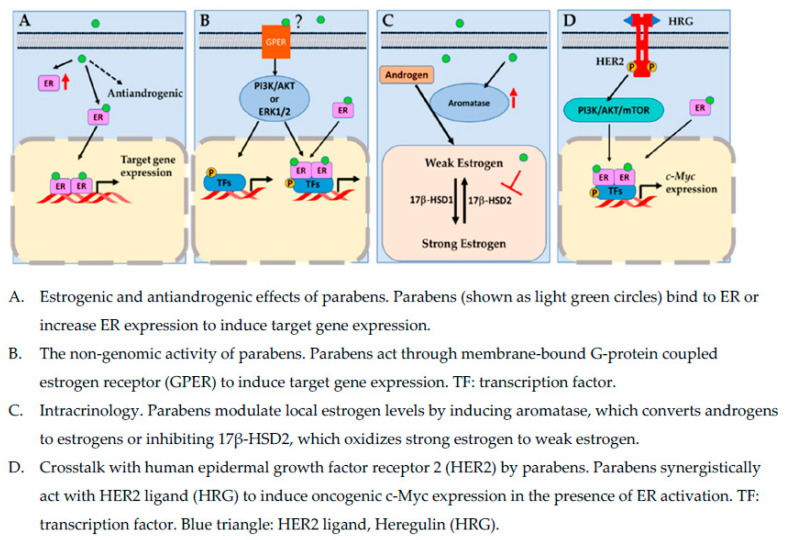
Molecular mechanisms by which parabens act in breast cancer cells.

Parabens are a group of EDCs commonly found in personal care products, foods, and pharmaceuticals. Systemic exposure to parabens has been confirmed by the ubiquitous detection of parabens in human blood and urine samples. The main concerns regarding parabens use in consumer products are their potential mimicry of endogenous hormones, possible cross-talks with other signal transduction pathways, such as HER2 signaling pathway, that are pivotal in the development of breast cancer, and modulation of key enzymes involved in local estrogen metabolism. Estrogen receptor is a key transcriptional factor that drives the oncogenesis and growth of hormonally sensitive breast cells. However, the recurrence and resistance to endocrine therapy of certain types of breast cancer, indicate that underlying mechanisms of breast cancer development are likely complex, involving multiple signaling pathways. Figure 1 highlights some possible mechanisms by which parabens affect breast cancer development.

Although some research findings have suggested that parabens may negatively interfere with some endocrine targets relevant to breast carcinogenesis, evidence from in vivo and epidemiological studies linking paraben exposure to breast cancer is limited. So far, most studies have focused on one single paraben and/or the direct modulating effects of parabens on sex hormone receptors. Parabens are known to have significantly lower binding affinities to estrogen receptors than their endogenous counterparts. Therefore, the direct link of the estrogenic effect of parabens to breast carcinogenesis is debatable.

Future studies investigating paraben mixtures and their cross-talks with other EDCs or signaling pathways both in vivo and in vitro are warranted. For now, cautions should be taken when individuals, including breast cancer patients or individuals with high risk of breast cancer, make the decisions on personal care products containing parabens. 

## Figures and Tables

**Table 1:** Summary of Human Studies on Paraben Exposure and Breast Cancer.

	Conclusions	Strength	Limitations
Darbre et al. 2004 [19].	Intact parabens were found in the human breast tumor tissues. MP was present at the highest level and represented 62% of the total parabens extracted from breast tumor tissues.	Demonstrated that after exposure, a proportion of the parabens can remain intact in human body tissues. The levels of parabens measured in this study were comparable to the levels of parabens used in in vitro studies, indicating the levels of parabens detected in breast tissues could induce estrogenic effects in the human breast.	Small sample size. Analytical blank values might contain parabens introduced from other sources.
Barr et al. 2011 [32].	Parabens were detected across the human breast from axilla to sternum. PP was found at significantly higher levels in the axilla than mid or medial regions of the breast. No correlations were found between paraben concentrations and tumor location or tumor estrogen receptor content.	Measured at the earliest time point possible after cancer diagnosis and prior to any cancer therapy. Investigated the distribution of parabens across a single breast.	Parabens were detected in breast tissues from human subjects who self-reported as non-underarm cosmetics users. Parabens were measured from breast tissues adjacent to breast tumor but not from tumor directly. It is also unclear the relative importance of long-term accumulation and/or acute exposure to the levels of parabens in the breast tissue.
Sprague et al. 2013 [34].	Serum concentrations of BP and PP were modestly correlated, but parabens concentrations were not associated with percentage breast density (a marker of breast cancer risk).	Evaluated mammographic breast density in relation to biological measures of xenoestrogens, including parabens. Serum measurements may better reflect the biologically relevant exposure of the target organs.	Only postmenopausal women were enrolled in the study. Single blood sample was collected, which may only reflect current exposure. The study population was predominately non-Hispanic white. The results may not apply to general population.
Harley, et al. 2019 [30].	Peripubertal concentrations of MP were associated with earlier breast development, pubic hair development, and menarche in girls; peripubertal concentrations of PP were associated with earlier pubic hair development in girls; peripubertal PP concentrations were associated with earlier genital development in boys.	Evaluated prenatal as well as peripubertal parabens exposure.	Urinary parabens only reflected recent exposure. The study population was limited to Latino women and children of low socioeconomic status. Potential confounding factors from other environmental contaminants could not be ruled out. Associations of peripubertal measurements with parabens may reflect reverse causality because children going through puberty earlier may be more likely to use personal care products.
Parada et al. 2020 [35].	The highest (vs. lowest) quintiles of urinary MP, PP, and total parabens were associated with the risk of breast cancer. MP, PP, and total parabens were inversely associated with all-cause mortality hazard ratios.	A case-control and follow up design. Large sample size. Participants included women with breast cancer and women without breast cancer. Among women with breast cancer, phenol biomarkers were quantified in urine samples. Women with breast cancer were monitored for vital status with a median follow-up of 17.6 years. Examined whether urinary phenol biomarkers were associated with mortality following breast cancer.	A single spot urine sample may not be a reliable reflection of women’s parabens exposure. In addition, urine samples from breast cancer patients were collected after not before their diagnosis.
Wu et al. 2021 [36].	Breast cancer risk was weakly inversely associated with total (but not individual) parabens exposure. Risk of hormone receptor positive (HR+) cancer was lower among women in the upper two tertiles of paraben exposure.	A multiethnic population-based nested case-control study. Examined the association between breast cancer risk and prediagnostic exposures paraben. Potential differences in terms of hormone receptor status, tumor stage (invasive vs in situ) and the length of follow-up time were considered in the analysis.	All cases and controls were postmenopausal at the time of urine collection. A single measure of parabens to capture long-term exposures could lead to misclassification of exposure.

**Table 2:** Effect of 17β-estradiol (E2) and Parabens on Estrogen and Progesterone Receptors Gene and Protein Expression [52].

	**MCF-7**							
	**ERα**				**PR**			
	**mRNA**		**Protein**		**mRNA**		**Protein**	
	**6 h**	**24 h**	**48 h**	**72 h**	**6 h**	**24 h**	**48 h**	**72 h**
MP	NC	↑	↑	↑	NC	NC	↑	↑
BP	NC	↑	↑	NC	NC	↑	NC	NC
PP	NC	↑	↑	NC	NC	↑	↑	↑
E2	NC	↑	↑	↑	↑	↑	↑	↑
	**MCF-10A**							
	**ERα**				**PR**			
	**mRNA**		**Protein**		**mRNA**		**Protein**	
	**6 h**	**24 h**	**48 h**	**72 h**	**6 h**	**24 h**	**48 h**	**72 h**
MP	↑	NC	↑	↑	NC	NC	NC	NC
BP	NC	NC	NC	NC	NC	↑	NC	NC
PP	↑	NC	NC	↑	NC	↑	NC	NC
E2	NC	↑	↑	↑	NC	NC	NC	NC
	**MCF-7**				**MCF-10A**			
	**ERβ**				**ERβ**			
	**mRNA**		**Protein**		**mRNA**		**Protein**	
	**6 h**	**24 h**	**48 h**	**72 h**	**6 h**	**24 h**	**48 h**	**72 h**
MP	NC	↑	↑	↑	NC	NC	NC	↑
BP	NC	↑	↑	NC	NC	NC	NC	↑
PP	NC	↑	↑	NC	NC	NC	NC	↑
E2	NC	↑	↑	↑	NC	NC	NC	NC

MCF-7 and MCF-10 A cells were exposed to parabens (20 nM) or E2 (10 nM); Gene expression was evaluated at 6 and 24 hours post treatment. Protein expression was evaluated at 48 and 72 hours post treatment. Up arrow: Upregulation; NC: No change.

**Table 3:** Effect of 17-β Estradiol (E2) and Parabens on the Expression of Selected Genes in MCF-7 and MCF-10A [57].

		**MCF-7**												
	**Genes**	**CCND1**	**CCND3**	**CCNE1**	**CCNE2**	**CCNA2**	**CDK2**	**CDK4**	**CDK6**	**CDKN1A**	**ATR**	**ATM**	**E2F3**	**TP53**
E2		↑	↑	NC	↑	↑	↑	↑	↓	↑	↑	↓	↓	NC
MP		NC	NC	↓	NC	NC	NC	NC	↓	NC	↑	↓	NC	NC
PP		NC	NC	↑	NC	NC	↑	NC	↓	NC	↑	↓	↓	NC
BP		NC	↑	↓	↑	NC	NC	NC	↓	NC	↑	↓	↓	NC
		**MCF-10A**												
	**Genes**	**CCND1**	**CCND3**	**CCNE1**	**CCNE2**	**CCNA2**	**CDK2**	**CDK4**	**CDK6**	**CDKN1A**	**ATR**	**ATM**	**E2F3**	**TP53**
E2		↑	NC	↑	↑	NC	↑	↑	NC	↓	NC	NC	↑	↑
MP		↑	NC	↑	NC	↑	↑	↑	↓	↓	NC	NC	↑	NC
PP		↑	↓	↑	NC	↑	↑	↑	NC	↓	NC	↑	↑	↑
BP		↑	↓	↑	↑	↑	↑	↑	↑	↓	↑	↑	↑	NC

MCF-7 and MCF-10A cells were exposed to MP, BP, and PP (20 nM) or E2 (10 nM) for 24 hours. Up arrow: Upregulation of gene expression; down arrow: Downregulation of gene expression; NC: Non change.

## Data Availability

Not applicable.

## Abbreviations

17β-HSD1	17β-hydroxysteroid dehydrogenases type 1
17β-HSD2	17β-hydroxysteroid dehydrogenases type 2
AR	Androgen receptor
ATM	Ataxia telangiectasia mutated
ATR	Ataxia telangiectasia and Rad3 related
BP	Butylparaben
BMI	Body mass index
BzP	Benzylparaben
CCND1	Cyclin D1
CCND3	Cyclin D3
CCNE1	Cyclin E1
CCNE2	Cyclin E2
CCNA2	Cyclin A2
CDK2	Cyclin Dependent Kinase 2
CDK4	Cyclin Dependent Kinase 4
CDK6	Cyclin Dependent Kinase 6
CDKN1A	Cyclin Dependent Kinase Inhibitor 1A
CYP19A1	cytochrome P450 19A1 (aromatase)
DHT	Dihydrotestosterone
E2F3	Transcription factor E2F3
EEF	Estradiol equivalence factor
E1	Estrone
E2	17β-estradiol
ER	Estrogen receptor
EDCs	Endocrine-disruptive chemicals
EMT	Epithelial-to-mesenchymal transitions
EP	Ethylparaben
GPER	G-protein-coupled estrogen receptor (GPR30 or GPER1)
GR	Glucocorticoid receptor
HeP	hexylparaben
HepP	Heptylparaben
HER2	Human epidermal growth factor receptor 2
HRG	Heregulin
IsoBP	Isobutylparaben
IsoPP	Isopropylparaben
MMPs	Matrix metalloproteinases
MP	Methylparaben
PhP	Phenylparaben
PR	Progesterone receptor
PP	Propylparaben
TP53	Tumor protein p53
